# Perspectives on a Way Forward to Implementation of Precision Medicine in Patients With Diabetic Kidney Disease; Results of a Stakeholder Consensus-Building Meeting

**DOI:** 10.3389/fphar.2021.662642

**Published:** 2021-05-04

**Authors:** Elisabeth Bakker, Peter G. M. Mol, João Nabais, Thorsten Vetter, Matthias Kretzler, John J. Nolan, Gert Mayer, Anna K. Sundgren, Hiddo J. L. Heerspink, Anja Schiel, Sieta T. de Vries, Maria F. Gomez, Friedrich Schulze, Dick de Zeeuw, Michelle J. Pena, for the BEAt-DKD Consortium

**Affiliations:** ^1^ Department of Clinical Pharmacy and Pharmacology, University of Groningen, University Medical Center Groningen, Groningen, Netherlands; ^2^ Dutch Medicines Evaluation Board (CBG-MEB), Utrecht, Netherlands; ^3^ Scientific Advice Working Party, European Medicines Agency (EMA), Amsterdam, Netherlands; ^4^ Associação Protetora Dos Diabéticos de Portugal, Lisboa, Portugal; ^5^ Comprehensive Health Reserach Centre (CHRC), Departamento de Ciências Médicas e da Saúde, Escola de Saúde e Desenvolvimento Humano, Universidade de Évora, Évora, Portugal; ^6^ European Medicines Agency (EMA), Amsterdam, Netherlands; ^7^ University of Michigan, Michigan Medicine, Internal Medicine/Nephrology and Computational Medicine and Bioinformatics, Ann Arbor, MI, United States; ^8^ University of Dublin, Trinity College, Dublin, Ireland; ^9^ Department of Internal Medicine IV (Nephrology and Hypertension), Medical University Innsbruck, Innsbruck, Austria; ^10^ AstraZeneca, Gaithersburg, MD, United States; ^11^ Norwegian Medicines Agency, Oslo, Norway; ^12^ Department of Clinical Sciences, Lund University, Diabetes Centre, Malmö, Sweden; ^13^ Boehringer Ingelheim International GmbH, Ingelheim, Germany

**Keywords:** precision medicine, personalized medicine, consensus meeting, diabetic nephropathy, diabetic kidney disease, stakeholder conference

## Abstract

**Aim:** This study aimed to identify from different stakeholders the benefits and obstacles of implementing precision medicine in diabetic kidney disease (DKD) and to build consensus about a way forward in order to treat, prevent, or even reverse this disease.

**Methods:** As part of an ongoing effort of moving implementation of precision medicine in DKD forward, a two-day consensus-building meeting was organized with different stakeholders involved in drug development and patient care in DKD, including patients, patient representatives, pharmaceutical industry, regulatory agencies representatives, health technology assessors, healthcare professionals, basic scientists, and clinical academic researchers. The meeting consisted of plenary presentations and discussions, and small group break-out sessions. Discussion topics were based on a symposium, focus groups and literature search. Benefits, obstacles and potential solutions toward implementing precision medicine were discussed. Results from the break-out sessions were presented in plenary and formed the basis of a broad consensus discussion to reach final conclusions. Throughout the meeting, participants answered several statement and open-ended questions on their mobile device, using a real-time online survey tool. Answers to the statement questions were analyzed descriptively. Results of the open-ended survey questions, the break-out sessions and the consensus discussion were analyzed qualitatively.

**Results and conclusion:** Seventy-one participants from 26 countries attended the consensus-building meeting in Amsterdam, April 2019. During the opening plenary on the first day, the participants agreed with the statement that precision medicine is the way forward in DKD (*n* = 57, median 90, IQR [75–100]). Lack of efficient tools for implementation in practice and generating robust data were identified as significant obstacles. The identified benefits, e.g., improvement of the benefit-risk ratio of treatment, offer substantive incentives to find solutions for the identified obstacles. Earlier and increased multi-stakeholder collaboration and specific training may provide solutions to alter clinical and regulatory guidelines that lie at the basis of both obstacles and solutions. At the end of the second day, the opinion of the participants toward precision medicine in DKD was somewhat more nuanced (*n* = 45, median 83, IQR [70–92]) and they concluded that precision medicine is *an* important way forward in improving the treatment of patients with DKD.

## Introduction

Interventions in the Renin-Angiotensin System (RAS), first the ACE inhibitors (ACEi) in 1993 and later the Angiotensin-Receptor Blockers (ARBs) in 2001, were breakthrough therapies to slow the progression of renal disease in patients with type 2 diabetes mellitus (Lewis et al., 1993; Nathan et al., 1993; Brenner et al., 2001; Lewis et al., 2001). However, despite this success, the residual renal and cardiovascular (CV) risk in this population remained extremely high (Heerspink and de Zeeuw, 2011). After the introduction of ACEi and ARBs, many new and combination therapies have been studied in large clinical trials including further RAS-blockade, as well as targeting other biological mechanisms, but these did not result in further renal protection in patients with type 2 diabetes mellitus, and sometimes even resulted in harm (Pfeffer et al., 2009; Mann et al., 2010; Packham et al., 2012; Parving et al., 2012; de Zeeuw et al., 2013; Fried et al., 2013). Only recently, the endothelin receptor antagonist atrasentan, the sodium glucose cotransporter 2 (SGLT2) inhibitors canagliflozin and dapagliflozin and the non-steroidal, selective mineralocorticoid receptor antagonist finerenone, studied in the SONAR, CREDENCE, DAPA-CKD, and the FIDELIO-DKD trials respectively, showed renal and CV protection (Heerspink et al., 2019; Perkovic et al., 2019; Bakris et al., 2020; Heerspink et al., 2020).

When re-analyzing the early positive trials of ACEi and ARBs, it appeared that the overall renal protection was mainly driven by efficacy in a specific group of patients, namely those in which the RAS-intervention reduced the albuminuria (de Zeeuw et al., 2004). Similarly, various post-hoc analyses of failed trials suggested that also in those trials a specific group of patients was protected by the investigational drug, namely those patients who showed responses in the targeted surrogate marker (de Zeeuw and Heerspink, 2016; Heerspink et al., 2016). Excluding those patients who showed harmful responses in biomarkers of renal damage could turn a failed trial into a renoprotective trial (Chin et al., 2014).

The trial failures and, consequently, the sustained unmet medical need for new diabetic kidney disease (DKD) treatments may have been the result of overlooking the variability in drug target response of individual patients that were recruited in the trials. Indeed, between-patient differences in albuminuria response and its potential effect on individual renal protection have been reported before (Laverman et al., 2002). For example, RAS-intervention is only protective in patients with a renal disease progression related to a mechanism that involves the RAS pathway (Parving et al., 2008). Other, non-responding, patients may need another therapeutic approach. The individual variability in response to treatment results in differences in effectiveness on the target, and differences in adverse and off-target effects. In drug development and authorization, these off-target effects are monitored for the safety of an intervention, but it is mostly ignored if they affect the intended surrogate and primary outcome positively or negatively. Renal and CV disease are often not driven by one single underlying mechanism, and patients also have different causes determining individual risk. Current evidence-based guidelines are based on results from large interventional trials, and although these have been key to the improvement of overall quality of care of DKD patients, they do not take into account the heterogeneity of the response in patients (de Vries et al., 2018; de Zeeuw and Heerspink, 2020).

The variability in response between patients to single drugs should be considered in both drug development and clinical practice: a drug that does not or not sufficiently modify the targeted risk factor will not protect the patient, and should not be prescribed. Moreover, such a drug may even harm the patient. A drug that lowers the risk marker and has no adverse effects is likely to protect the patient and/or to improve the quality of life. In analogy to the treatment practice of choosing a specific antibiotic for an infected patient, and selecting a specific drug for a cancer patient based on a tumor biopsy, both examples of fields in which precision medicine is already widely used, the treatment modifying renal and CV risk in DKD should be tailored to the individual patient. Therefore, DKD treatment development should abandon the idea of “one size fits all” and instead focus on “a personalized fit” (de Zeeuw and Heerspink, 2020).

The European Biomarker Enterprise to Attack DKD (BEAt-DKD) consortium is one of several large consortia that are investigating several aspects of this precision medicine approach in DKD. The consortium comprises 24 academic institutions and clinics, seven big pharma European Federation of Pharmaceutical Industries and Associations (EFPIA) partners, two small and medium enterprises and one patient organization (www.beat-dkd.eu). This consortium seeks to optimize the renoprotective treatment of patients with DKD by studying the actual disease mechanism, detecting the determinants and individual variability of drug response, and identifying biomarkers to allow implementation of these findings in practice leading to optimized and more precise medicine.

In the precision medicine approach, many different stakeholders are involved, including patients and patient advocates, basic scientists and clinical academic researchers (hereafter referred to as “scientists”), pharmaceutical industry representatives (hereafter referred to as “industry”), pharmaceutical regulators (hereafter referred to as “regulators”), health technology assessors (HTAs) and healthcare professionals (HCPs). All these stakeholders have different backgrounds and expertise and, therefore, have their own perspectives on introducing precision medicine in DKD. Delineating these perspectives by bringing stakeholders together drives the discussion on possible ways forward toward implementation of precision medicine in DKD. The BEAt-DKD consortium aimed to identify from these different stakeholders’ individual and mutual benefits and obstacles of implementing precision medicine and to define solutions to overcome the obstacles. The aim was to build consensus about a way forward to implement precision medicine in treating, preventing, halting, or even reversing DKD.

## Methods

**FIGURE 1:**
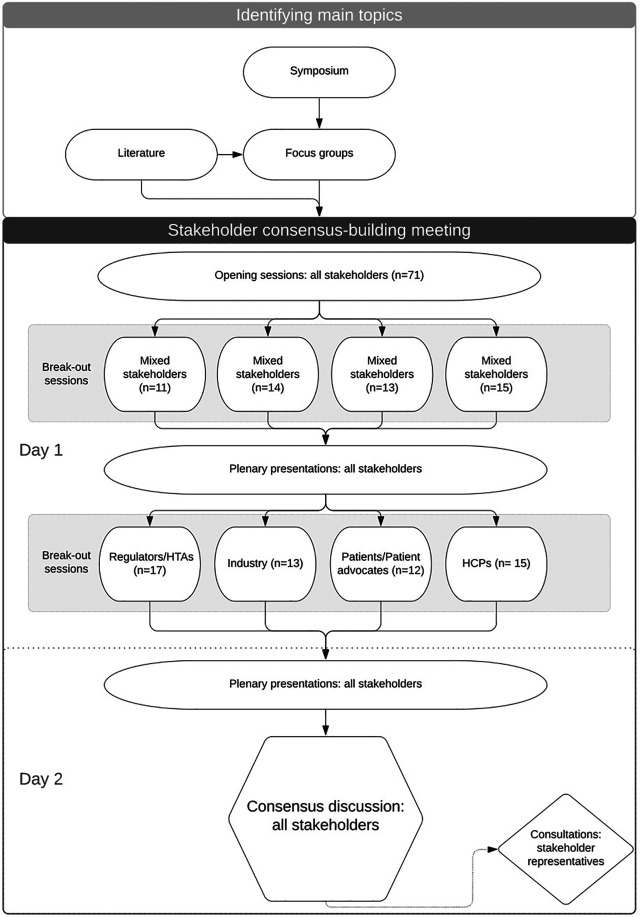
Flowchart of the process of building consensus on a way forward to implementation of precision medicine in diabetic kidney disease during the consensus-building meeting in Amsterdam, April 2019. HTA, health technology assessors; HCPs, healthcare professionals (“n” equals the number of participants of the survey).

In order to build consensus on a way forward to implement precision medicine in DKD, a series of meetings were organized over 2017–2018: a symposium, small-scale focus group sessions and a consensus-building meeting (Figure 1). In the first step, in a more traditional symposium, various speakers outlined their perspectives on precision medicine in DKD. The topics discussed during this symposium were elaborated on in a series of articles published in a special issue on precision medicine in DKD (de Vries et al., 2018; Heerspink and de Zeeuw, 2018; Heerspink et al., 2018; Mol et al., 2018; Mulder et al., 2018).

In the second step, stakeholder specific focus group discussions were organized with representatives of European regulatory agencies (*n* = 7), HTAs (*n* = 4), HCPs (*n* = 5), patients (*n* = 4) and BEAt-DKD partners (*n* = 10) that expanded on these perspectives on precision medicine in DKD. During these focus group discussions a background presentation on precision medicine in DKD was given, and thereafter a moderated semi-structured discussion was held. General and stakeholder-specific topic lists were developed by BEAt-DKD members. The symposium and focus group results combined with a literature search led to the selection of the main topics for an interactive consensus-building meeting. Main topics included costs, feasibility, evaluation aspects and acceptability to patients.

The consensus-building meeting was held in Amsterdam, the Netherlands, on April 3–4, 2019. For this consensus-building meeting, we invited experts in the fields of pharmaceutical industry, European HTAs and regulators, including representatives from the Dutch Medicines Evaluation Board (MEB), the European Medicines Agency (EMA) and the US Food and Drug Administration (FDA), and scientists. To identify patient associations and HCP groups, we performed web searches and used contacts from the European Association for the Study of Diabetes (EASD) and the network of BEAt-DKD members. Potential participants were contacted by e-mail.

The two-day consensus-building meeting consisted of several sessions (Supplementary File S1) and participants completed short surveys with questions based on the previously identified topics. For the short surveys, a real-time online survey tool (Mentimeter, www.mentimeter.com) was used and all participants could answer each question individually on their mobile device.

The first day of the consensus-building meeting was structured by having a mixture of plenary presentations by speakers representing the different stakeholder groups and discussions, and two small group break-out sessions with discussions once with mixed stakeholder groups and once per stakeholder group (Figure 1).

### Opening Plenary

During the opening plenary, the survey tool was used to ask all participants for their consent, their self-reported stakeholder designation and to ask how much they agreed on the statement “Precision medicine is the way forward in DKD” on a visual analogue scale ranging from 1–disagree to 100–agree.

### Break-Out Session 1: Mixed Stakeholder Groups

For the first break-out session, stakeholders were divided into four groups by the organizers of the meeting to have mixed stakeholder groups. Participants’ stakeholder designation was based on self-reporting. During the session, three open-ended questions were used in the survey tool to identify major benefits and obstacles of precision medicine for patients with DKD and tentative solutions to overcome these obstacles. The aim of this first break-out session was that the participants got acquainted with perspectives on precision medicine of other stakeholders.

### Break-Out Session 2: Individual Stakeholder Groups

During the second break-out session, participants were assigned to the individual stakeholder groups by the organizers of the meeting based on their professions. Due to the smaller number of regulators and HTAs, these groups were combined into one group. Scientists were divided among the stakeholder groups. In this second break-out session, participants were asked to answer 13 statements on Likert scales (11 statements with 5-point Likert scales that ranged from 1 = strongly disagree to 5 = strongly agree, one with a 5-point Likert scale that ranged from 1 = minor concern to 5 = major concern and one with a 7-point Likert scale that ranged from 1 = totally unacceptable to 7 = perfectly acceptable). These statements represented the previously identified main topics for the various stakeholder groups. In addition, one open-ended question and one multiple answer question was asked (Supplementary File S2). One to five questions per stakeholder group were focused on that specific stakeholder group but they were asked to all the stakeholders.

In both break-out sessions, the answers given by the participants were discussed in the groups. These discussions were led by a member of the BEAt-DKD consortium.

### Day 2: Plenary Presentations and Consensus Discussion

The second day, a summary of findings on the benefits, obstacles and solutions of the second break-out session on the first day per stakeholder group was presented by the moderators of the sessions. These formed the basis for the broad consensus discussion that followed to draw mutual conclusions. At the end of the meeting, stakeholders were asked again how much they agreed on the statement “Precision medicine is the way forward in DKD” on a visual analogue scale ranging from 1–disagree to 100–agree.

After the general program, consultations with a selected group of representatives of these stakeholder groups lead to a common agenda on issues to address in implementing precision medicine in DKD. This agenda is based on the five main questions that were covered in the consensus discussion: 1) Are you in favor of precision medicine in DKD? 2) What are the benefits of precision medicine in DKD? 3) What are the obstacles of precision medicine in DKD? 4) What are possible solutions? 5) What is the practical way forward?

Answers to the open-ended questions in the survey, and the discussion results of the break-out sessions and consensus discussion were analyzed qualitatively by one researcher (EB) and reviewed by a second researcher (MP). The Likert-scale data of the survey are presented descriptively. The results are presented according to the five main questions in the common agenda and are supported by quotes from the answers given to the open-ended questions.

## Results and Discussion

Approximately 150 individuals were invited, and 71 participants from 26 countries attended the consensus-building meeting, mostly from European countries, e.g., Germany, the Netherlands, Spain, Sweden and United Kingdom, but also from the United States, Asia and Australia. These 71 participants consisted of 23 scientists, including nine PhD candidates and students, seven regulators, e.g., clinical assessors and scientific officers from EMA, FDA, and national regulatory agencies, 16 HCPs, e.g., nephrologists, endocrinologists, and clinical pharmacists, five HTAs, ten industry representatives from seven different pharmaceutical companies, and ten patients/patient advocates from diabetes or kidney associations.

### Are You in Favor of Precision Medicine in Diabetic Kidney Disease?

**FIGURE 2:**
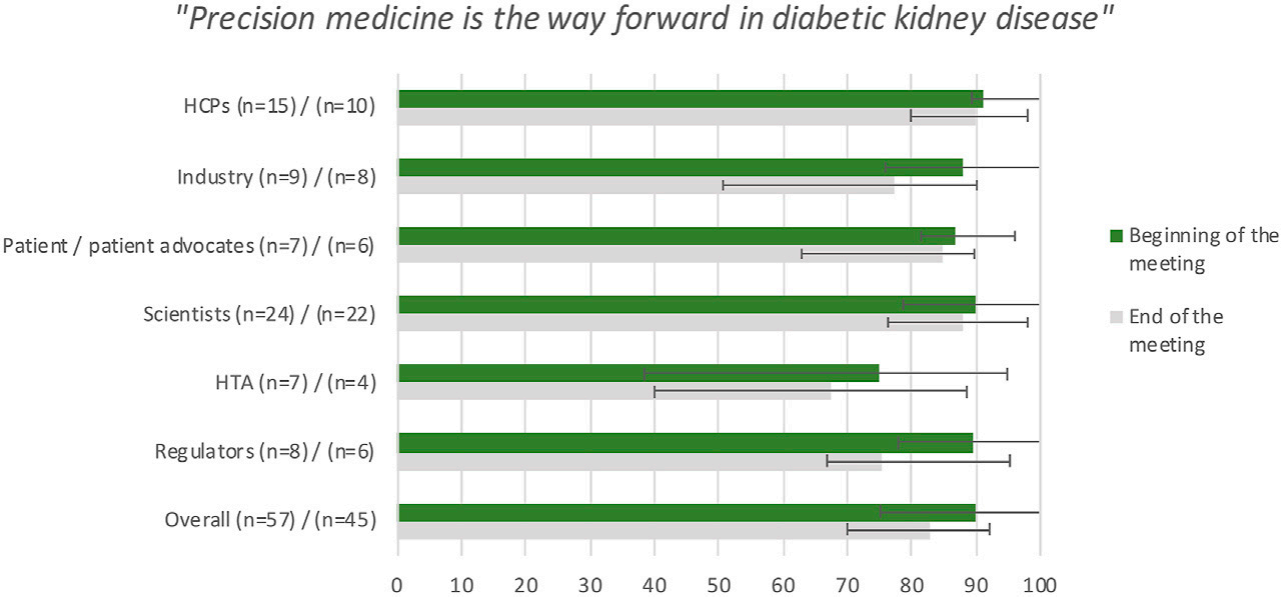
Median and interquartile range of the answers per stakeholder group to the question “How much do you agree with the statement below: Precision medicine is the way forward in Diabetic Kidney Disease?” on a Visual Analogue Scale ranging from 0 (disagree)–100 (agree) at the beginning and the end of the two-day meeting. (HCP, healthcare professional; HTA, health technology assessor).

During the opening plenary on the first day, the participants indicated that precision medicine is the way forward in DKD (*n* = 57, median 90, IQR [75–100]). The median responses ranged from 75 for HTAs to 91 for HCPs (Figure 2).

The joint opinions per stakeholder group of the second break-out session that were reported back to the plenary session revealed that the stakeholder groups agreed that precision medicine is *a* way forward for diabetes, rather than being *the* way forward as was perhaps implied on the first day of the meeting. Answers to the statement “Precision medicine is the way forward in DKD” were still positive at the end of the meeting (*n* = 45, median 83, IQR [70–92]), but slightly less than it was during the opening plenary (Figure 2). This slight decrease may have been caused by getting acquainted with the obstacles and complexity of implementing precision medicine from different stakeholders’ perspectives. During the consensus discussion patients/patient advocates recommended a more cautious formulation of the statement to avoid creating false hope and it was agreed that the statement could have been formulated more accurately as “Precision medicine is *a* way forward to find the right treatment for the right patient at the right time”, as precision medicine could certainly be used to optimize the treatment for every individual.

### What are the Benefits of Precision Medicine in Diabetic Kidney Disease?

Identified benefits were mainly related to optimization of treatment. This primarily included increased effectiveness of drugs and less harms–i.e., a better benefit-risk ratio. Better selection of the right patients and, therefore, avoiding prescription of ineffective, unnecessary and/or even harmful drugs and thus avoiding both over- and under treatment were considered important benefits.

*“Identify precisely those patients with high absolute risk”, “Identifying patients at need”* and *“Differentiation of drugs in terms of treatment response (individual benefit-risk)”* (industry)

*“Better stratification of patients might allow optimized organization of care”* (HCP)

*“Less side effects”,* and *“Better outcomes”* (patients/patient advocates)

Furthermore, precision medicine may improve adherence and quality of life of patients, e.g., by taking into account patient preferences and improving patients’ perspectives.

*“PM (Precision Medicine) has the potential to increase drug adherence”* and *“PM might allow better to include important endpoints like QOL”* (HCPs)

Some stakeholders expected the cost-effectiveness of drugs to be better as well. One example of this is expectations regarding savings in the drug development phase.

*“Smaller studies, larger effect size”* (industry)

*“Bring studies back to more manageable sizes”* (regulator/HTA)

*“Faster drug development”* (patient/patient advocate)

**FIGURE 3:**
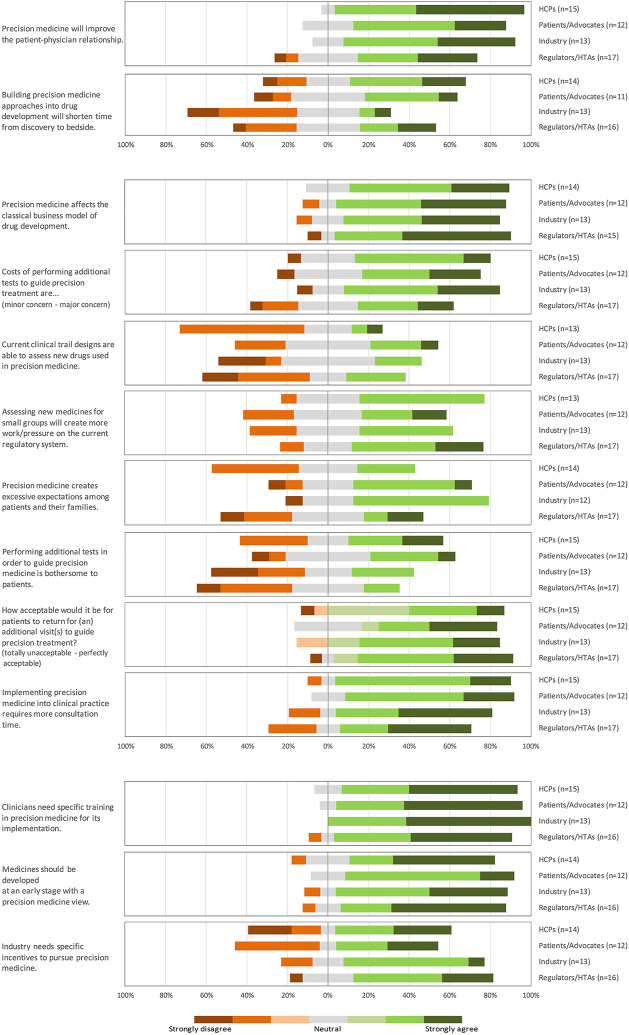
Results of survey: stakeholder opinions of precision medicine. (HCPs, healthcare professionals; HTAs, health technology assessors).

The results of the statements in the survey show that the stakeholders believed that precision medicine could improve the patient-physician relationship (median 4, IQR [4–5]) (Figure 3), via e.g., more frequent visits and shared decision making. The HCPs thought that precision medicine will shorten the time to get a drug from discovery to bedside in DKD (median 4, IQR [3–4]), whereas industry disagreed (median 2, IQR [2–3]) and other stakeholder groups were rather neutral (median 3, IQR [2.5–4]). Responses to the open question why they thought precision medicine will or will not shorten the time from discovery to bedside in DKD varied from *“Targeted, so benefit may be seen earlier, so shorter and smaller trials”* (HCP) to *“It depends”* (several stakeholders) and *“We don’t know - we have no empiric evidence one way or another. The counterfactual is also uncertain”* (Patient/patient advocate) to *“No, more complicated trials/evaluation”* (regulator/HTA).

### What Are the Obstacles of Precision Medicine in Diabetic Kidney Disease?

The proposed obstacles were a bit more diverse. Most obstacles related to the implementation of precision medicine.

*“Patients are in clinical routine not characterized and tracked well enough to predict risks precisely”* and *“Diagnosis rates are already today with the available armamentarium low”* (industry)

*“Just because evidence is there it does not automatically get implemented in daily clinical care”* (patient/patient advocate)

*“Precision medicine in DKD has to run up against a well-established, seemingly very effective and safe standard of care”* (HCP)

Precision medicine can only happen if suitable conditions for implementation could be realized and adequate data will be generated. Specifically, regulators and HTAs worried that robust data is hard to obtain, which is a requirement for benefit-risk and cost-effectiveness decision making. Both industry and HCPs called precision medicine “*very aspirational*”’ and “*now only realistic in subjects at greatest risk or non-responding to the standard of care*”. HCPs were in doubt if precision medicine should be applied to all patients or if the focus should be on non-responders to currently available therapies.

“*Practice will change. The tests could be complicated and time consuming as will be the issue of short-term repetitive examination of patients to adjust therapy.”* (HCP)

Most stakeholders were concerned about costs, in contrast with the better cost-effectiveness listed as benefits. Analogous to the implementation obstacles, stakeholders were concerned that clinical studies are more difficult to design, partly because validated/qualified biomarkers and other tools are still lacking.

*“Need for new diagnostics that must be validated/certified”* and *“The price of implementation of biomarker tests”* (regulator/HTA)

*“Defining cut-offs for response/no response and agreeing this with regulators”* and *“Qualification and validation of biomarkers and surrogates and the workload coming along with it”* (industry)

The results of the statements in the survey show that stakeholders thought that precision medicine will affect the classical business model of drug development (median 4, IQR [4–5]) (Figure 3) and increased costs were quite a concern (median 4, IQR [3–4], ranging from 1 = minor concern, 5 = major concern). Stakeholders were unsure whether current clinical trial designs are adequate to assess new drugs used in precision medicine (median 3, IQR [2–3.5]). Precision medicine may result in multiple smaller trials in smaller treatment groups, which may lead to multiple applications that regulators/HTAs think may increase workloads (median 4, IQR [3–4]), whereas other stakeholders were less sure regulators’ and HTAs’ workloads would increase. Patients/patient advocates and industry thought that precision medicine creates excessive expectations among patients and their families (both median 4, IQR [3–4]), other stakeholders were more neutral (HCPs: median 3, IQR [2–3.75]; regulators/HTAs: median 3, IQR [2–4]). From literature and the earlier conducted focus groups, additional testing and additional doctor appointments were proposed obstacles (Weldon et al., 2012; Rothstein, 2017). Interestingly, stakeholders were neutral toward how bothersome they thought additional tests would be to patients (median 3, IQR [2–4]), even the patients themselves had no strong feelings about this (median 3, IQR [3–4]). The stakeholders found it (rather) acceptable for patients to have one or more additional doctor appointments to guide precision treatment (median 6, IQR [5–6] of 7-point Likert scale). Stakeholders did think that precision medicine in clinical practice would take more consultation time (median 4, IQR [4-5]).

### What Are Possible Solutions?

Solutions suggested by stakeholders varied widely. Most stakeholders advocated for more (international) cooperation between all stakeholders, e.g., working in consortia/working groups, more engagement from patients and industry, or guidance from regulators/HTAs. An integrated healthcare system based on precision medicine was considered a solution as well.

*“Early interaction with regulators/HTAs on drug development/trial design”* (regulator/HTA)

*“Alignment of stakeholders, i.e., regulators across the Atlantic”* (industry)

*“Involve regulators in research from day 1”,* and *“Use data and target politicians, payer and decision makers.”* (patients/patient advocates)

*“HCPs have to work as a team.”* and *“Database systems have to be aligned to allow easy access to all necessary biomarkers”* (HCPs)

Exploring new companion diagnostics, biomarkers, study designs and new tools, such as statistical methods or artificial intelligence qualified and accepted for use in drug development were proposed solutions to provide scientific proof of precision medicine’s promise.

*“Newer types of trials: platform trials and factorial designs”* (regulator/HTA)

*“Switch from on-top-of and parallel group hard outcome studies to surrogate endpoints, cross-over, head-to-head trials to identify the patients benefitting most from a drug”* (industry)

Finally, stakeholders thought that both patients and HCPs should receive further education about precision medicine.

*“Education of physicians/HCP and patients”* (industry)

*“We have to educate colleagues and patients”* (HCP)

The latter was also shown in the results of the statement in the survey that clinicians need specific training in precision medicine for its implementation (median 5, IQR [4–5]) (Figure 3). Also, the survey results show that medicines should be developed at an early stage with a precision medicine view (median 4, IQR [4–5]). Most regulators/HTAs and industry at the meeting agreed that industry needs specific incentives to pursue precision medicine (median 4, IQR [3–4.25]; median 4, IQR [3–4] respectively) but patients/patient advocates and HCPs fluctuated more in their answers (median 3.5, IQR [2–4.25]; median 4, IQR [2–4.75] respectively).

### What Is the Practical Way Forward?

According to the patients/patient advocates, obtaining robust data as evidence for precision medicine’s effectiveness requires involvement of each stakeholder. This importance is also addressed in the recently published consensus report for precision medicine in diabetes from the American Diabetes Association (ADA) and the European Association for the Study of Diabetes (EASD) (Chung et al., 2020), and is consistent with a report reviewing the 15th Annual Precision Medicine Conference (Wells, 2020) stressing that *“No stakeholder can do it by itself”.* They concluded that key stakeholders should share data and learn from it to support efficient refinement and implementation of more sophisticated precision medicine strategies and to drive future progress in healthcare (Wells, 2020).

Furthermore, the patients/patient advocates indicated that for them thresholds should be clear to set personal goals, e.g., reduced risks or increased quality of life. Defined outcomes should certainly include quality of life, not only hard clinical outcomes. The other stakeholders shared the same vision; answers to the question “How should you assess if precision medicine is effective?” showed that quality of life and patient-reported outcomes are an important assessment read-out for new precision-based medicines. Perhaps surprisingly, quality of life was rated more important than clinical events by all stakeholders (overall 82 vs. 75%). According to the HCPs, risk stratification vs. response-to-treatment-based stratification in clinical studies should be clearly defined. Proper communication and introduction of precision medicine to patients is required. It was important for HCPs to identify the non-responders to standard of care. This calls for education and HCP collaboration and, possibly, using tools, such as machine learning to identify patients that may benefit from therapy (Belur Nagaraj et al., 2020). Eventually, a precision medicine-to-standard of care comparison is desired for all aspects and endpoints.

Industry stakeholders endorsed the importance of identifying non-responders and developing new drugs for these non-responders. Regulators/HTAs agreed with other stakeholders on the importance of determining what happens to the non-responders and deciding on relevant thresholds. Therefore, response biomarkers and a good understanding of thresholds of risk and response are needed in both care and trial design. Trials including a subset of a biomarker-negative population as well as a biomarker-positive population could provide a solution. Current care should be evaluated on these aspects. Furthermore, current practice of reimbursement should be adapted. Costs of implementing biomarkers and added care should be considered when setting prices of precision medicine drugs. New trial designs were considered desirable, as long as they are able to generate robust data required for regulatory assessment. It was mentioned that a difficulty with DKD trial design specifically may be that the best renal care solution may not be the best CV care solution. This was shown in for example the BEACON and the SONAR trial, in which the treatment groups showed higher risk of adverse CV effects compared to placebo (Chin et al., 2014; Bakris et al., 2020).

### Future Perspectives

Efforts like the organized symposium, the focus groups and the consensus-building meeting, contribute to the implementation of precision medicine in DKD. Although the concrete, direct impact of the meetings is not clear, several stakeholders present at our meeting also attended the broader consensus meeting on precision medicine in diabetes from the American Diabetes Association (ADA) and the European Association for the Study of Diabetes (EASD). This may well lead to building further partnerships and initiatives, as they aimed for in their future perspectives (Chung et al., 2020). In a more general precision medicine context, EMA has integrated the stimulation of developments in precision medicine and the role of biomarkers as an important goal of the Regulatory Science Strategy to 2025 (European Medicines Agency, 2020). In this view, several organizers of the consensus-building meeting have organized an expert meeting as being part of the Regulatory Science Network Netherlands (RSNN), in which several additional research gaps focusing on robust evidence generation and regulatory assessment were discussed (Regulatory Science Network Netherlands, 2020). Implementation of precision medicine into drug development and clinical practice moves slowly, and our series of meetings, including the consensus-building meeting, are therefore part of an ongoing effort to stimulate this with the ultimate goal to improve treatment for each individual patient.

### Limitations

The findings of the consensus-building process should be seen in consideration of some limitations. The views expressed in this meeting represent individuals and are not necessarily shared by the larger community or the organization they work for. Participants of a precision medicine consensus-building meeting may already have an interest in precision medicine and therefore attended the meeting or they may have already attended previous meetings on this topic. This may have biased some of the results including the high positive perspective toward precision medicine in DKD. The individual stakeholder break-out session groups did not consist exclusively of the specific stakeholders, but were combined and supplemented with scientists. Also, during the plenary session and first break-out session the self-reported stakeholder group designation might have been different from the stakeholder group designation of the second break-out session. Finally, the regulator/HTA group comprised stakeholders from only a few, mainly European, countries. It should be noted that in some countries the regulatory body and the HTA body are separate, whereas in other countries they operate within one organization. As the regulatory and HTA bodies do not operate in similar health care systems, the results may not be representative for different countries in- and outside Europe. Individual stakeholder group results should therefore be interpreted with caution.

## Conclusion

Implementing precision medicine is one solution to tackle the high unmet need of new DKD treatment. A clear definition of precision medicine and its different components and requirements is needed to warrant all stakeholders’ commitment and to implement precision medicine successfully. Stakeholders in the drug development trajectory for DKD indicated that there are still many obstacles that slow effective implementation in practice. However, the envisaged benefits offer clearly a great incentive to find solutions for the defined obstacles. This will require multi-stakeholder involvement, training, collaboration and initiatives to alter both regulatory and clinical practice policies and guidelines that lie at the basis of both obstacles and solutions. To implement precision medicine successfully, early engagement and aligning stakeholders’ goals are critical. This collaboration should be maintained throughout the drug development cycle both within and between stakeholder groups.

## Data Availability

The data used for this study are available upon reasonable request to the corresponding author.
