# Supplementary material for: Perspectives on a Way Forward to Implementation of Precision Medicine in Patients With Diabetic Kidney Disease; Results of a Stakeholder Consensus-Building Meeting
Source: Front Pharmacol. 2021 May 4;12:662642. doi: 10.3389/fphar.2021.662642 (PMC8132196; doi:10.3389/fphar.2021.662642)
Supplement: Supplementary file 1 [file DataSheet1.pdf]

Supplementary files

S1. Programme of the Consensus-building Meeting on Precision Medicine in Diabetic Kidney Disease; April 3 - 4, 2019

<u>Day 1 - Wednesday, April 3 2019</u>

09:00 – 09:30 Welcome and goal of the symposium – *Maria Gomez, Lead BEAt-DKD, Dick de Zeeuw, Lead WP6*

09:30 – 10:45 Break out 1, Mixed stakeholder groups

11:00 – 11:30 Patient's view on precision medicine – *João Nabais, University of Evora and Associação Protetora dos Diabéticos de Portugal*

11:30 – 12:00 Precision medicine in primary care – *Xavier Cos, Primary Care Diabetes Europe*

12:00 – 12:30 How will we pay for precision medicine – *Anja Schiel*, *Norwegian Medicines Agency*

13:30 – 14:00 Developing new drugs for precision medicine – *Anna Sundgren, AstraZeneca*

14:00 – 14:30 Regulatory approval of precision medicines – *Thorsten Vetter, EMA*

14:30 – 15:00 Regulatory approval of precision medicines – *Aliza Thompson, FDA*

15:45 – 16:15 Biomarkers for precision medicine in DKD – *Matthias Kretzler, University of Michigan*

16:15 – 16:45 Improving outcomes with precision medicine – *John Nolan, European Diabetes Forum*

16:45 – 18:00 Break out 2, Individual stakeholder groups

Day 2 - Thursday, April 4 2019

08:30 – 09:00 Welcome and key points from Day 1 – *Friedrich Schulze, Boehringer Ingelheim*

09:00 – 10:00 Groups report back to plenary

10:30 – 12:00 Consensus discussion – *Dick de Zeeuw, Lead WP6*

S2. List of Survey Questions

Opening plenary

1. Which stakeholder perspective do you represent today at the symposium?
2. How much do you agree with the statement below?: Precision medicine is the way forward in Diabetic Kidney Disease.

Break-out session 1: mixed stakeholder groups

3. Which stakeholder perspective do you represent today at the symposium?
4. How much do you agree with the statement below?: Precision medicine is the way forward in Diabetic Kidney Disease.
5. What is the biggest benefit of precision medicine?
6. What is the biggest obstacle of precision medicine?
7. What are specific solutions to overcome these obstacles?

Break-out session 2: individual stakeholder groups

Industry

8. How much do you agree with the following statement?: Medicines should be developed at an early stage with a precision medicine view.
9. How much do you agree with the following statement?: Precision medicine affects the classical business model of drug development.
10. How much do you agree with the following statement?: Industry needs specific incentives to pursue precision medicine.
11. How much do you agree with the following statement?: Building precision medicine approaches into drug development will shorten time from discovery to bedside.
12. Building precision medicine approaches into drug development will shorten the time from discovery to bedside: yes or no - why? (open question)

Regulators

13. How much do you agree with the following statement?: Assessing new medicines for small groups will create more work/pressure on the current regulatory system.
14. How much do you agree with the following statement?: Current clinical trial designs are able to assess new drugs used in precision medicine.

HTAs

15. How much do you agree with the following statement?: Costs of performing additional tests to guide precision treatment are... (minor concern – major concern, 5-point Likert scale).
16. How should you assess if precision medicine is effective? (multiple answer)
    a. Real World to Clinical trial
    b. Quality of Life
    c. Patient Reported Outcomes
    d. Surrogates (e.g., blood pressure, glucose, albuminuria)
    e. Clinical events (e.g., heart attack, stroke, dialysis, transplantation)
    f. Safety outcomes

Patients

17. How much do you agree with the following statement?: Performing additional tests in order to guide precision medicine is bothersome to patients.
18. How acceptable would it be for patients to return for (an) additional visit(s) to guide precision treatment? (totally unacceptable – very acceptable, 7-point Likert scale)
19. How much do you agree with the following statement?: Precision medicine creates excessive expectations among patients and their families.

HCPs

20. How much do you agree with the following statement?: Clinicians need specific training in precision medicine for its implementation.
21. How much do you agree with the following statement?: Implementing precision medicine into clinical practice requires more consultation time.
22. How much do you agree with the following statement?: Precision medicine will improve patient-physician relationship.

Consensus-building discussion

23. Which stakeholder perspective do you represent today at the symposium?
24. How much do you agree with the statement below?: Precision medicine is the way forward in Diabetic Kidney Disease.
